# Development for generating electric power shoes having a vibrating sheet generator assembly

**DOI:** 10.1186/1757-1146-7-S1-A95

**Published:** 2014-04-08

**Authors:** Jia Hroung Wu, Wen Lan Wu

**Affiliations:** 1 Department of Industrial Management, Hsiuping University of Science and Technology, Taichung City, Taiwan; 2 Department of Sports Medicine, Kaohsiung Medical University, Kaohsiung City, Taiwan

## 

It is often seen an object move or vibrate repeatedly. Some phenomenons are useful, but another is unavailable motion. The unavailable motion makes a lot of energy dissipation. In order to utilize the energy unavailable motion makes, the research will use the energy to generate electric power for solving the deficient personal energy sources problems. In general, the generator coil sweeps the magnetic field line to generate the electric power these days. The revolving spindle will abrasion and the energy will loss. Therefore, the generating efficiency of electric power will decrease [1-4].

The research utilizes a structure of sheet generator to generate electric power. The present research relates to a method for manufacturing a sheet generator having a flat coil assembly, and more particularly to a method comprising the steps of placing a flat coil into an injecting mold; forming a locating section to secure the flat coil; and assembling the sheet generator. The relative motion between coil and magnet will generate electric power in accordance with the Fleming’s right-hand rule.

In order to assess the efficiency of generating electric power, the research will design an experimental device to simulate the sheet generator. First, two plastic diaphragms be used to laminate the coil. Then, place the laminate diaphragm on the inverted U-shape structure of experimental device. Because the inverted U-shape structure and magnet move relatively, so the electric power will be produce by cutting magnetic field line.

**Figure 1:**
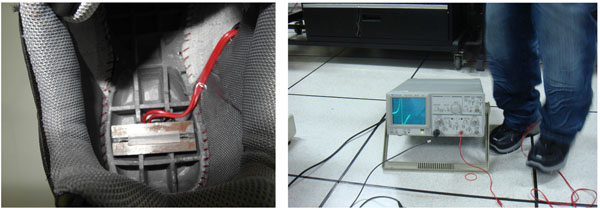
Testers wore shoes for generating voltage measurements

The server motor drives the cam to press the inverted U-shape structure. When the motor rotational speed is 120 r.p.m. (2Hz), then the voltage of generating electric power can obtain exceeds respectively 1.5V, 2.0V and 2.5V without assembling the bridge circuit at the coil of 1 layer, 2 layers and 3 layers, as shown in Table 1. Actually, the voltages of generating electric power shoes exceed respectively 1V and 2V without assembling the bridge circuit under the human walking motion and running motion, as shown in Figure 1. The basic goal of the research has achieved. And the design parameters can easily provide the industry of electric power shoes. The industry of storage energy will be developed and established.

**Table 1:** The table of relationship between number of coil layers and voltage

Layers	1	2	3
Resistance (Ω)	29.2	62.5	92.2

Voltage (V)	1.5	2.5	3.0
